# The sugar transporter SWEET10 acts downstream of *FLOWERING LOCUS T* during floral transition of *Arabidopsis thaliana*

**DOI:** 10.1186/s12870-020-2266-0

**Published:** 2020-02-03

**Authors:** Fernando Andrés, Atsuko Kinoshita, Naveen Kalluri, Virginia Fernández, Vítor S. Falavigna, Tiago M. D. Cruz, Seonghoe Jang, Yasutaka Chiba, Mitsunori Seo, Tabea Mettler-Altmann, Bruno Huettel, George Coupland

**Affiliations:** 1 Max Planck Institute for Plant Breeding Research, Carl-von-Linne-Weg 10, 50829 Köln, Germany; 2 Present Address: UMR AGAP, Univ. Montpellier, INRAE, CIRAD, INSAAE, Montpellier, France; 3 Present Address: BPMP, Univ Montpellier, CNRS, INRAE, Montpellier SupAgro, Montpellier, France; 4 Present Address: World Vegetable Center Korea Office (WKO), 100 Nongsaengmyeong-ro, Iseo-myeon, Wanju-gun, Jellabuk-do 55365 Republic of Korea; 5 RIKEN Center for Sustainable Resource Science, 1-7-22 Suehiro-cho, Tsurumi-ku, Yokohama, Kanagawa 230-0045 Japan; 6 Cluster of Excellence on Plant Sciences and Institute of Plant Biochemistry, Heinrich-Heine University, 40225 Düsseldorf, Germany

**Keywords:** Flowering time, FLOWERING LOCUS T, Photoperiod, Sugar transporter

## Abstract

**Background:**

Floral transition initiates reproductive development of plants and occurs in response to environmental and endogenous signals. In *Arabidopsis thaliana*, this process is accelerated by several environmental cues, including exposure to long days. The photoperiod-dependent promotion of flowering involves the transcriptional induction of *FLOWERING LOCUS T* (*FT*) in the phloem of the leaf. *FT* encodes a mobile protein that is transported from the leaves to the shoot apical meristem, where it forms part of a regulatory complex that induces flowering. Whether FT also has biological functions in leaves of wild-type plants remains unclear.

**Results:**

In order to address this issue, we first studied the leaf transcriptomic changes associated with FT overexpression in the companion cells of the phloem. We found that FT induces the transcription of *SWEET10*, which encodes a bidirectional sucrose transporter, specifically in the leaf veins. Moreover, *SWEET10* is transcriptionally activated by long photoperiods, and this activation depends on FT and one of its earliest target genes *SUPPRESSOR OF CONSTANS OVEREXPRESSION 1* (*SOC1*). The ectopic expression of *SWEET10* causes early flowering and leads to higher levels of transcription of flowering-time related genes in the shoot apex.

**Conclusions:**

Collectively, our results suggest that the FT-signaling pathway activates the transcription of a sucrose uptake/efflux carrier during floral transition, indicating that it alters the metabolism of flowering plants as well as reprogramming the transcription of floral regulators in the shoot meristem.

## Background

In plants, the transition from vegetative growth to flowering is regulated by several environmental and endogenous stimuli. This complexity is conferred by a network of genetic pathways that has been characterized in most detail in the model species *A. thaliana*. This network includes the vernalisation, gibberellin, thermosensory, age, sugar and photoperiod-dependent pathways [1–3]. *FLOWERING LOCUS T* (*FT*) is a positive regulator of flowering whose expression leads to rapid transcriptional reprogramming of the meristem associated with inflorescence and flower development, and is often described as a floral integrator because its transcription is activated by several genetic pathways that promote flowering [1, 4, 5]. Sugars such as sucrose and trehalose-6-phosphate also promote flowering, and there is evidence that these act both upstream and downstream of *FT* in the flowering process [6–9]. However, our understanding of the relationship between FT and sugar metabolism is fragmentary. Here, we demonstrate that FT is required for the transcriptional activation of a gene encoding a sugar uptake/efflux carrier in the vasculature of the leaf and at the shoot apex during floral transition, providing a specific link between FT function and sugar transport.

*FT* was first placed within the photoperiodic flowering pathway of *A. thaliana* based on physiological and genetic analyses [10]. Furthermore, simultaneous loss-of-function of *FT* and its closest relative *TWIN SISTER OF FT* (*TSF*) leads to late flowering plants under long days (LDs) that are almost insensitive to photoperiod [11, 12]. Transcription of *FT* is induced by exposure to LDs downstream of the *GIGANTEA* (*GI*) and *CONSTANS* (*CO*) genes in specialized companion cells of the phloem [13–16]. *FT* encodes a small globular protein that shares high homology with mammalian phosphatidylethanolamine-binding proteins (PEBP) [4, 5], and is a major component of a systemic signal that induces flowering in response to photoperiod (a “florigen”) [17–20]. FT protein moves through the phloem to the shoot apical meristem (SAM) by an active mechanism [21, 22] and binds lipids in vitro [23]. An endoplasmic reticulum-membrane protein, FT-INTERACTING PROTEIN 1 (FTIP1), interacts with FT in companion cells of the phloem and mediates its export into sieve elements [21]. In the sieve elements, FT interacts with a heavy metal-associated isoprenylated plant protein called SODIUM POTASSIUM ROOT DEFECTIVE 1 (NaKR1), which regulates the long-distance transport of FT to the SAM [22]. In the SAM, FT is proposed to interact with two bZIP transcription factors (FD and FD PARALOG [FDP]) [24–26]. The transcriptional complex that is formed between FT, these bZIPs and 14–3-3 proteins is proposed to trigger transcriptional activation of genes that promote flowering, such as *SUPPRESSOR OF OVEREXPRESSION OF CONSTANS* 1 (*SOC1*), *FRUITFULL* (*FUL*) and *APETALA1* (*AP1*), which encode MADS-box transcription factors, and several members of the *SQUAMOSA PROMOTER BINDING LIKE* (*SPL*) gene family [24, 25, 27–30]. Transcriptomic and in situ hybridization studies identified *SOC1* mRNA as the earliest activated transcript detected in the SAM during *FT*-mediated photoperiodic induction of flowering [28, 29, 31–33], while genome-wide experiments showed that SOC1 binds to the promoters of numerous genes involved in the floral transition and floral meristem identity [34, 35]. Therefore, SOC1 acts as an intermediate component in the FT-signaling pathway during the activation of flowering of *A. thaliana*. Consistently, the flowering response to *FT* overexpression is attenuated in the *soc1* single mutant [36], and this effect is even more pronounced in the *soc1 ful* double mutant [29, 36].

Ectopic expression of *FT* from heterologous promoters leads to early flowering [4, 5]. For example, overexpression of *FT* from constitutive promoters such as the *Cauliflower mosaic virus CaMV 35S* promoter [p*35S*] [4, 5] or phloem-specific promoters such as those of the *GALACTINOL SYNTHASE1* [*GAS1*] and *SUCROSE TRANSPORTER2* [*SUC2*] genes [17, 19, 20, 37] induces early flowering of *A. thaliana*. This effect is highly conserved among Angiosperms, so that overexpression of *FT* or its homologues causes early flowering in a wide range of species [18, 38, 39]. Overexpression of *FT* also induces the transcription of *FUL* and *SEPALLATA3* (*SEP3*) in leaves of *A. thaliana*, conferring changes in leaf morphology that are suppressed by *ful* and *sep3* mutations [40]. Thus, at least when overexpressed, FT can influence the development of leaves by affecting the expression of regulatory genes, and upon transport from the leaves it promotes the floral transition at the SAM.

Here, we have further studied the regulatory role of FT. We analyzed global transcriptomic changes in leaves associated with the specific expression of *FT* in the phloem companion cells. Our results indicate that *FT* promotes the expression of *SWEET10*, a gene encoding a sucrose bidirectional transporter, in the leaf veins and at the shoot apex. This effect is also mediated by photoperiod and by *SOC1*. Moreover, the overexpression of *SWEET10* slightly accelerates flowering, leading us to discuss possible roles for this gene during floral transition mediated by the FT-signaling pathway in *A. thaliana*.

## Results

### *FT* induces the expression of *SWEET10*

**Fig. 1:**
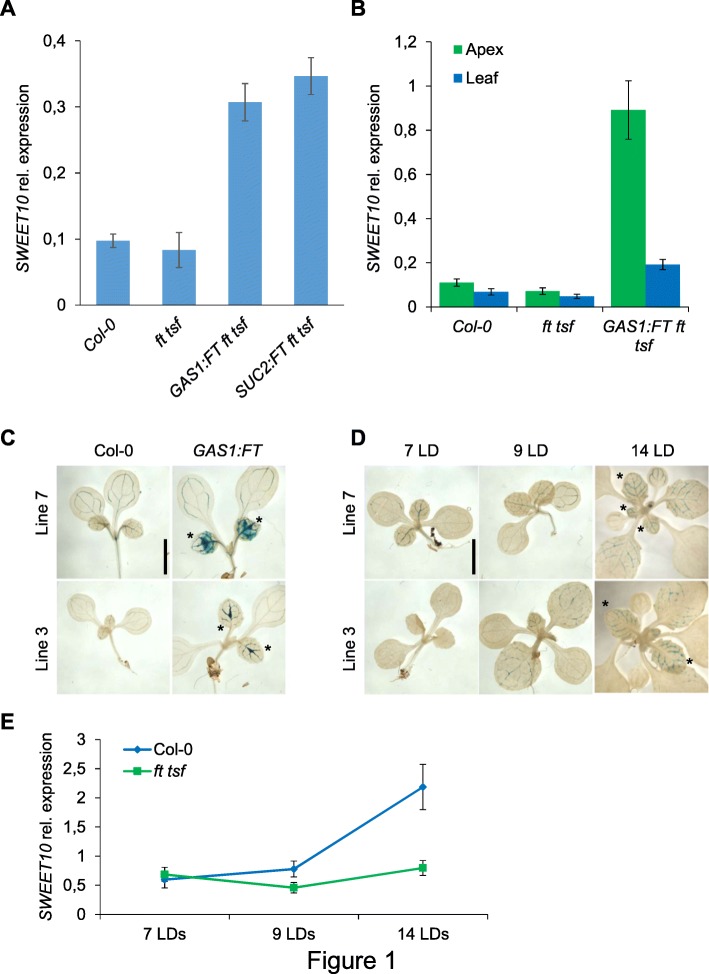
*FT* activates the transcription of *SWEET10* mRNA. **a** Quantification of *SWEET10* mRNA levels in leaves of different *FT*-overexpressing plants (9 LDs at ZT16). **b** Comparison of *SWEET10* mRNA levels in leaves and shoot apices of different *FT*-overexpressing plants (9 LDs at ZT16). **c** *GUS* expression levels in T3 p*SWEET10:GUS* (Col-0) independent transgenic lines #3 and #7 (left) and in T1 plants from the cross between p*GAS1:FT* and p*SWEET10:GUS* lines #3 and #7 (right) at 7 LDs. **d** GUS staining of p*SWEET10:GUS* transgenic lines #3 and #7 in Col-0 background (T3 generation) during a time-course in LDs. Pictures of (**c**) and (**d**) were taken at ZT8. Scale bar = 5 mm. Asterisks indicate young leaves. **e** Quantification of *SWEET10* expression levels in shoot apices of Col-0 and *ft tsf* double mutants in a time-course under LDs. Shoot apices were sampled at ZT8. Errors bars in A, B and E indicate Standard Deviation

The global effects on gene expression of *FT* overexpression in the phloem companion cells of the leaves were examined. To this end, transgenic *A. thaliana* plants that overexpress *FT* from the *pGAS1* promoter in a *ft − 10 tsf − 1* double mutant background were employed (p*GAS1:FT ft − 10 tsf-1*). In these transgenic plants, the use of the p*GAS1* promoter ensures that the *FT* transgene is expressed in phloem companion cells of the minor veins, recreating the spatial pattern of expression described for the native gene [15]. Indeed, the overexpression of *FT* from the p*GAS1* promoter complements the late-flowering phenotype of *ft-10 tsf-1* double mutants [11, 17]. The transcriptome of leaves of p*GAS1:FT ft-10 tsf-1* transgenic plants was compared to that of *ft-10 tsf-1* and Col-0 plants using Tiling Arrays. Bioinformatic analysis showed that 699 genes (*p*-Value ≤0.01) were differentially expressed between p*GAS1:FT ft-10 tsf-1* and *ft-10 tsf-1* (Additional file 1)*.* A final list of 14 genes (Table 1) was selected by applying more restrictive statistical criteria (adj. *P*. Value ≤0.05). The majority of these genes were well-known flowering-related regulators that act downstream of FT [28, 29], such as *SOC1*, *FUL*, *SEPALLATA1* (*SEP1*) and *SEP3*, which were up-regulated in p*GAS1:FT ft-10 tsf-1*. In addition, *SWEET10* and *SWEET13*, two members of Clade III of the SWEET family that encode sucrose transporters [41], were in the list of genes induced by FT (Table 1). In particular, the mRNA levels of *SWEET10* were strongly up-regulated in the p*GAS1:FT ft-10 tsf-1* and other genetic backgrounds overexpressing *FT* (Fig. 1a). The expression of *SWEET10* mRNA was clearly induced in plants overexpressing *FT* from companion cell specific promoters pG*AS1* and p*SUC2* [42, 43] (Fig. 1a). This experiment demonstrated that in all cases tested, *FT* overexpression increased transcription of *SWEET10* mRNA. Additionally, in silico gene co-expression analyses indicated that *FT* is highly co-regulated with *SWEET10* during Arabidopsis development (Additional file 2: Figure S1A). These analyses also showed that *SWEET10* is co-expressed with many other genes that are regulated by FT (Additional file 2: Figure S1B), such as *SEP3* and *APETALA1* (*AP1*) [40].

**Table 1:** Top 14 differentially expressed genes between p*GAS1:FT ft-10 tsf-1* and *ft-10 tsf-1*

ID	logFC	*P*. Value	adj.P.Val	Gene Name
AT1G65480	6,03	4E-10	7,8E-06	*FT* (*FLOWERING LOCUS T*)
AT5G60910	2,83	2E-09	2,5E-05	*FUL* (*FRUITFULL*)
AT1G24260	2,66	8E-09	6E-05	*SEP3* (*SEPALLATA3*)
AT5G50790	2,28	2E-07	0,00144	*SWEET10*
AT2G45660	2,25	5E-10	7,8E-06	*SOC1* (*SUPPRESSOR OF OVEREXPRESSION OF CO 1*)
AT5G15800	1,92	4E-06	0,01516	*SEP1* (*SEPALLATA1*)
AT1G80130	1,24	2E-05	0,03677	*Tetratricopeptide repeat (TPR)-like superfamily protein*
AT5G50800	1,09	8E-06	0,02541	*SWEET13*
AT3G58200	0,82	1E-05	0,02772	*TRAF-like family protein*
AT3G56080	0,78	2E-05	0,04354	*S-adenosyl-L-methionine-dependent methyltransferases superfamily protein*
AT1G62290	-1,18	8E-06	0,02541	*PASPA2* (*PUTATIVE ASPARTIC PROTEINASE A2*)
AT5G44400	-1,31	1E-05	0,02772	*ATBBE26* (*BERBERINE BRIDGE ENZYME-LIKE 26*)
AT5G23020	-1,52	3E-06	0,01516	*IMS2/MAM-L/MAM3* (*METHYLTHIOALKYMALATE SYNTHASE-LIKE*)
AT2G42540	-3,21	9E-06	0,02541	*COR15A* (*COLD-REGULATED 15A*)

### *FT* promotes the expression of *SWEET10* mRNA in the leaf veins

Next, FT-mediated spatial and temporal regulation of *SWEET10* mRNA expression was characterized. The levels of *SWEET10* mRNA in leaves and hand-dissected shoot apices (containing SAM, a segment of the apical stem and young leaves) were quantified by RT-qPCR. As shown in Fig. 1b, *SWEET10* expression levels were higher in shoot apices compared to mature leaves and this difference was more pronounced in p*GAS1:FT ft-10 tsf-1* plants. To better characterize the spatial pattern of expression of *SWEET10*, we fused the 3 Kb region 5′ of the translational start codon to a *GUS* reporter gene to create p*SWEET10:GUS*. In transgenic plants harboring this reporter, GUS signal was restricted to the leaf veins and notably enhanced in young leaves of p*GAS1:FT* plants (Fig. 1c). Changes in p*SWEET10:GUS* expression were also studied during the floral transition under LDs. To this end, we monitored GUS signal in p*SWEET10:GUS* transgenic plants grown under LDs for 7, 9 and 14 days, the time window during which the floral transition occurs in our growth conditions. As observed in the previous experiment, the GUS signal was restricted to the leaf vasculature and it was stronger in young leaves (Fig. 1d). Furthermore, the GUS signal was more evident in plants undergoing the transition to reproductive phase (i.e. 9 to 14 days) compared to those at vegetative stage (i.e. 7 days) (Fig. 1d). This result was confirmed by a RT-qPCR experiment performed during the same time-course, in which an increase of *SWEET10* mRNA expression was observed in shoot apices containing young leaves of Col-0 plants from day 9 (Fig. 1e). The increase of *SWEET10* expression was largely suppressed in the *ft-10 tsf-1* mutant (Fig. 1 e). This indicates that the up-regulation of *SWEET10* during the floral transition of *A. thaliana* partially depends on the presence of a functional *FT* allele.

### The photoperiodic flowering pathway of *A. thaliana* regulates *SWEET10* expression

**Fig. 2:**
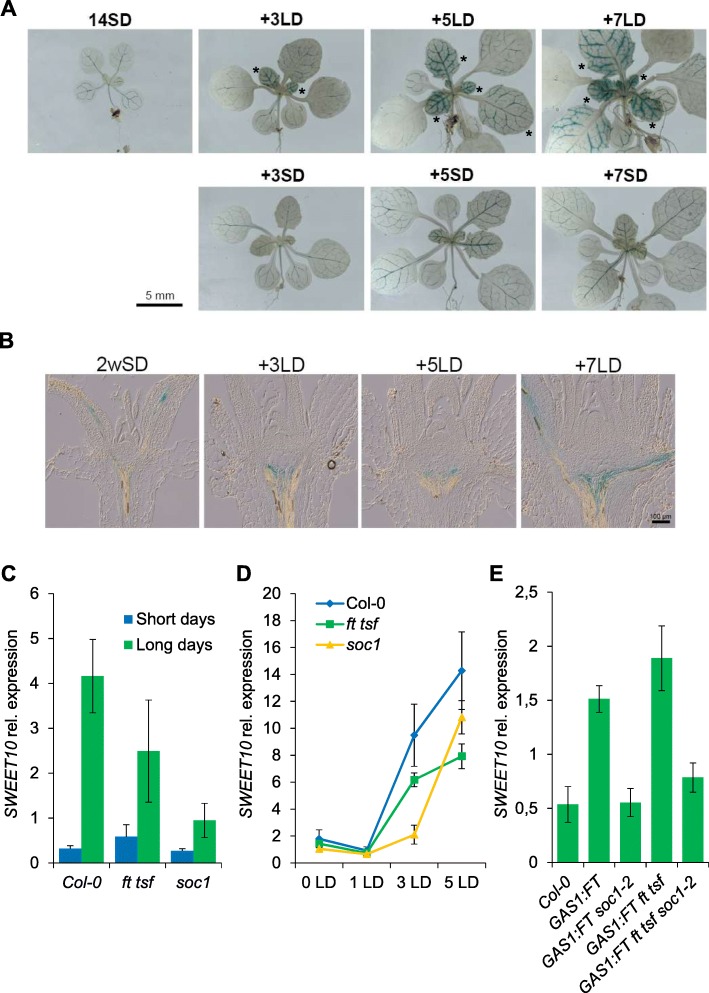
Photoperiod signaling pathway contributes to *SWEET10* mRNA induction. **a** and **b** GUS expression in plants expressing p*SWEET10:GUS* (line #7, T4 generation) grown under SDs for 2 weeks, shifted to LDs and collected for GUS staining at ZT8 after exposure to different numbers of long days. **a** Whole seedlings. Scale bar = 5 mm. Asterisks indicate young leaves. **b** Shoot apices were dissected and visualized under an optical microscope (× 20). Bar scale: 100 μm. **c**, **d** and **e** shows the expression levels of *SWEET10* mRNA in shoot apices of different genotypes at ZT8. In (**c**), plants were grown under LDs and SDs. In (**d**), plants were grown under SDs for 2 weeks, shifted to LDs shoot apices were harvested at ZT8 in different days. In (**e**), plants were grown under LDs and shoot apices sampled at ZT8. Errors bars in **c**, **d** and **e** indicate Standard Deviation

FT is a major component of the photoperiodic flowering pathway that promotes floral induction of *A. thaliana* in response to LDs. As the above experiments suggest that FT regulates *SWEET10* mRNA expression levels during floral transition, we tested whether the photoperiodic pathway activates *SWEET10* transcription. The expression of *GUS* in p*SWEET10:GUS* was monitored in plants grown under SDs and then shifted to LDs for 3, 5 and 7 days. An increase in intensity of the GUS signal was observed in the vascular tissue of leaves shifted to LDs compared to those grown under SDs (Fig. 2a), indicating that the *SWEET10* promoter responds to LDs. Furthermore, in cross-sections of the shoot apex of *pSWEET10:GUS* plants, *GUS* expression increased in the mature vascular tissue at the apex of plants shifted to LDs (Fig. 2b). In agreement with these observations, RT-qPCR analysis demonstrated that the levels of *SWEET10* mRNA were higher in plants grown under LDs compared to SDs (Fig. 2c) or after the shift of SD-grown plants to LDs (Fig. 2d). Interestingly, the *GUS* expression disappeared from leaves of *pSWEET10:GUS* plants at the end of the flowering phase. Instead, GUS expression was observed in reproductive organs, including anthers and siliques (Additional file 2: Figure S2). The photoperiod-dependent up-regulation of *SWEET10* mRNA also involves FT, because it is reduced in *ft-10 tsf-1* mutant plants (Fig. 2c and d). S*OC1* is a key component of the photoperiod signaling pathway that acts immediately downstream of FT [31, 44]. Therefore, whether *SWEET10* transcriptional regulation requires SOC1 downstream of FT was investigated. Remarkably, *SWEET10* mRNA levels were strongly reduced in a null mutant allele of *SOC1* (*soc1–2*) (Fig. 2d and e). Moreover, the introduction of the *soc1–2* mutation into transgenic plants overexpressing *FT* from the *GAS1* promoter was sufficient to largely suppress the enhanced transcriptional induction of *SWEET10* mediated by higher levels of FT (Fig. 2e). Collectively, these results indicate that *SWEET10* is transcriptionally regulated by the photoperiodic flowering pathway and this regulation involves the activities of *FT* and *SOC1*.

### Overexpression of *SWEET10* causes early flowering and affects the expression levels of genes that promote floral induction

**Fig. 3:**
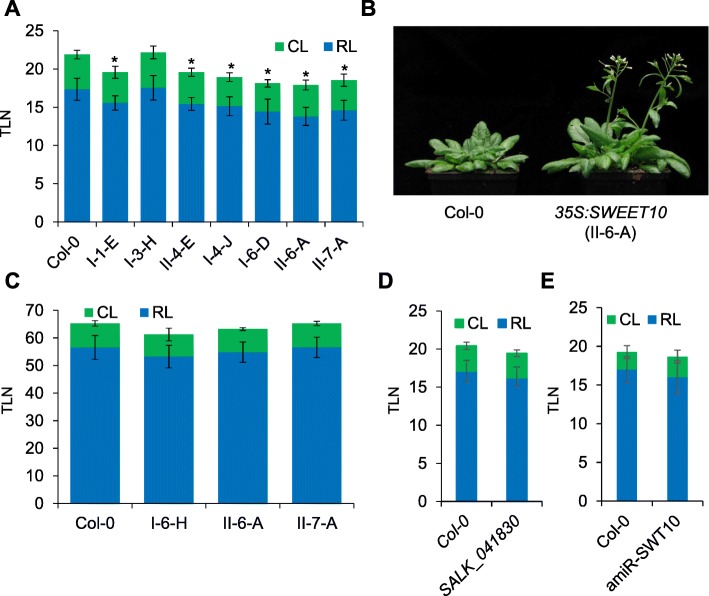
Overexpression of *SWEET10* promotes flowering under LDs. Flowering time of transgenic plants overexpressing *SWEET10* from the *35S* promoter under LDs (**a**) and (**b**), and under SDs (**c**). **d** Flowering time of the SALK_041830 T-DNA line compared to Col-0 under LDs. **e** Flowering time of a T3 transgenic line (#17–3) silencing *SWEET10* gene expression compared to Col-0 under LDs. At least 10 plants were used for each experiment. Asterisk indicates a significant difference compared to Col-0 (T-test, *p*-Value ≤0.05). TLN: Total Leaf Number; RL: Rosette Leaf number; CL: Cauline Leaf number. Errors bars in A, C, D and E indicate Standard Deviation

The results presented so far suggest that *SWEET10* transcription is induced by FT-signaling pathway via SOC1. In order to explore this possibility, we overexpressed *SWEET10* in *A. thaliana* plants and evaluated its effect on flowering time. We obtained several T1 transgenic lines that ectopically expressed *SWEET10* from the *35S* promoter (p*35S:SWEET10*). A higher level of *SWEET10* mRNA expression was observed for several of these lines compared to the control Col-0 lines (Additional file 2: Figure S3). We scored the flowering-time of homozygous single copy T3 transgenic lines. Seven out of 8 tested independent transgenic lines displayed a significant acceleration of flowering compared to the control plants under LDs (Fig. 3a and b). We also overexpressed *SWEET10* in the companion cells of the phloem from the *SUC2* promoter. However, most of the p*SUC2:SWEET10* transgenic plants did not flower earlier than the controls (Additional file 2: Figure S4). To address whether the overexpression of *SWEET10* could accelerate flowering independently of the photoperiodic pathway, we scored the flowering time of p*35S:SWEET10* plants under SD conditions. Under these conditions, p*35S:SWEET10* transformants flowered at similar times to the controls (Fig. 3c). This result suggests that the acceleration of flowering mediated by increased *SWEET10* mRNA levels requires LDs. Therefore, the flowering function of *SWEET10* could also depend on FT function. To further characterize the function of *SWEET10*, a T-DNA insertion line and transgenic plants expressing an artificial microRNA (amiR) that targets *SWEET10* mRNA were employed. None of these genetic backgrounds displayed a significant change in flowering time compared to the wild-type plants (Fig. 3d and e; and Additional file 2: Figure S5).

**Fig. 4:**
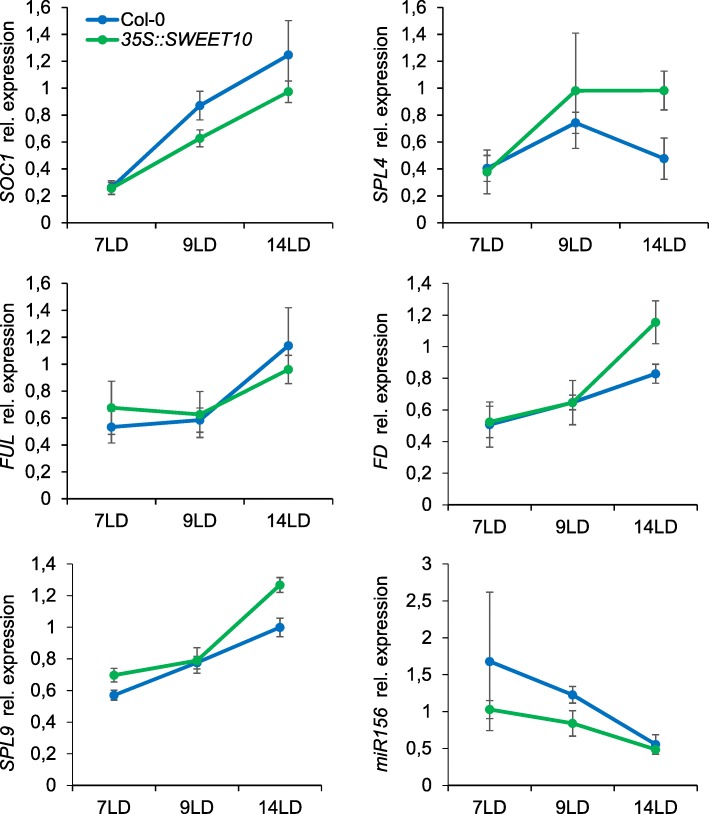
Expression levels of flowering-time related genes in *SWEET10* overexpressing plants. The expression levels of flowering-time related genes was quantified in Col-0 and p*35S:SWEET10* transgenic plants (Line II-6-A, T4 generation) under LDs. Shoot apices were collected at ZT8. Errors bars indicate Standard Deviation

In order to clarify the nature of the effect of *SWEET10* overexpression on flowering time, the expression levels of key regulators of flowering in *A. thaliana* were quantified in plants overexpressing *SWEET10* (Fig. 4). In this analysis, the mRNA levels of *FD* and some *SPL* genes (*SPL4* and *9*) were higher in shoot apices of p*35S:SWEET10* during reproductive development (14 LDs). This pattern of expression correlates with a possible role of *SWEET10* in promoting flowering. However, *SOC1* expression was slightly lower in p*35S:SWEET10* transgenics compared wild-type plants, whereas *FUL* mRNA levels were not differentially expressed at this developmental stage. Notably, the expression level of one precursor of miR156 (*MIRNA156C)*, which targets several mRNAs encoding SPLs, was reduced after 7 and 9 LDs in the p*35S:SWEET10* compared to wild type plants.

### *SWEET10* might be the only member of the *SWEET* family involved in the FT-flowering pathway in *A. thaliana*

**Fig. 5:**
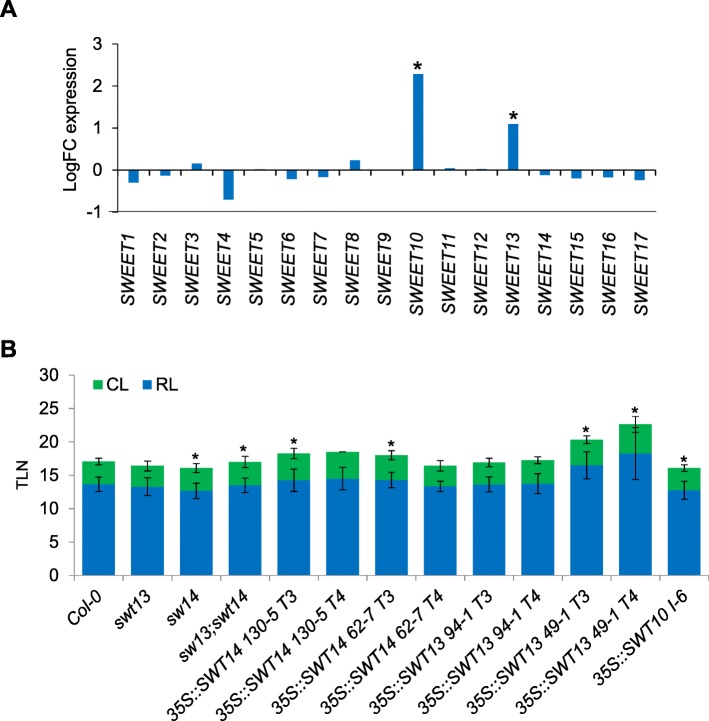
Involvement of *SWEET* family members the FT-flowering pathway. **a** Expression of *SWEET* family members in *GAS1:FT ft tsf* plants. The logFC between *GAS1:FT ft tsf* and Col-0 is represented. Significant differences are indicated with an asterisk (adj. *P*. Value ≤0.05). **b** Effect of the overexpression of *SWEET13* and *14* in flowering-time under LDs. At least 10 plants were used for each experiment. Asterisk indicates a significant difference compared to Col-0 (T-test, *p*-Value ≤0.05). Scale bar = 5 mm. Errors bars in B indicate Standard Deviation

*SWEET10* belongs to a large family of genes composed by 17 members in *A. thaliana* [45]. At least two of them, *SWEET10* and *13*, were deregulated in the microarray experiment that we performed (Table 1). We extracted the expression data of all 17 members of the family from the microarray analysis (Additional file 1). As shown in Fig. 5a, only the mRNA levels of *SWEET10* and *13* were significantly affected in p*GAS1:FT ft-10 tsf-1* plants compared to *ft-10 tsf-1* double mutants. Furthermore, we made use of promoter:*GUS* fusions to monitor the spatial and temporal expression of some other Clade III *SWEET* genes (*SWEET11*, *12*, *13* and *14*). In all the tested transgenic plants the *GUS* signal was detected in the vasculature under SDs, but did not increase after exposure to LDs, as was observed for p*SWEET10:GUS* (Fig. 2a and Additional file 2: Figure S6). Moreover, plants overexpressing *SWEET13* and *SWEET14* did not show acceleration of flowering under LDs. Instead, some of the tested lines displayed late flowering compared to the wild-type plants (Fig. 5b).

### Measurement of concentrations of fructose, glucose and sucrose at shoot apices during floral transition

The increased expression of *SWEET10* at the shoot apex during floral transition (Fig. 2), suggested that sugar levels might increase in this tissue during the transition. Therefore, concentrations of sucrose, fructose and glucose were tested in shoot apices harvested from plants grown for 2 weeks under short days and then transferred to LDs for 7 days to induce the transition. Although *SWEET10* expression increases under these conditions (Fig. 2d), no significant change in concentration of any of the sugars was detected across the time course (Additional file 2: Figure S7). Also, there was no significant difference in levels of these sugars between Col-0 and *ft tsf* genotypes (Additional file 2: Figure S7). Thus, based on this analytical method, no changes in sugar levels that correlated with the floral transition could be detected in apical samples.

## Discussion

Here, we demonstrate that *FT* and *SOC1* activate the transcription of the *SWEET10* gene. The pattern of transcription of *SWEET10* and the effect of its overexpression suggests that the sugar transporter it encodes contribute to the floral transition in *A. thaliana* under LDs. SWEET10 represents a previously undescribed link between sugar transport and the photoperiod-dependent control of flowering time.

### The transcriptional activation of *SWEET10* might be part of a genetic network controlled by the FT-pathway in leaves

FT protein is expressed in the vascular tissue of leaves and is transported to the shoot apex as a component of the florigenic signal that activates flowering [17, 19, 20]. However, whether FT has additional roles in the vascular tissue or in leaves remains unclear. Furthermore, overexpression of *FT* from the constitutive *35S* promoter caused the transcriptional induction in leaves of *FUL* and *SEP3*, which in wild-type plants are activated by FT signaling at the shoot apex [40]. Thus, we reasoned that transcriptomic analysis of plants overexpressing *FT* from a promoter specific to companion cells of the phloem, the cell type in which *FT* is expressed [15, 37], could identify additional genes that respond to FT function in wild-type plants. The majority of genes identified by this approach as significantly regulated by FT were already known to act downstream of FT during the floral transition and flower development, such as *SOC1, FUL, SEP1* and *SEP3* (Table 1). In addition, *SWEET10* was one of the most significantly differentially expressed genes in leaves of *GAS1:FT* plants and was co-regulated with these flowering genes (Additional file 2: Figure S1), suggesting that it could be activated in leaves by FT along with other known floral regulators.

FT is proposed to activate gene expression directly by interacting with the bZIP transcription factor FD that is expressed in the shoot meristem [24, 25]. However, it could also activate expression of genes such as *SWEET10* indirectly through the action of downstream transcription factors. We have shown that SOC1 regulates the transcription of *SWEET10* (Fig. 2d and e). This regulation would probably occur in the leaves, as *SOC1* is also expressed in these organs [46]. Thus, *SWEET10* is placed downstream of FT and SOC1, within a genetic network that operates in the leaves.

### Spatial-temporal distribution of *SWEET10* mRNA

Several *SWEET* genes belonging to Clade III (e.g. *SWEET11*, *12*, *13* and *14*) have been shown to be expressed in the vascular tissue of *A. thaliana*, most likely in the phloem [41, 47] (Chen et al., 2011; Kanno et al., 2016). In particular, *SWEET11* and *12* are expressed in specialized cells that form files along the veins and probably correspond to phloem parenchyma cells [41]. *SWEET10* was also expressed in the phloem (Fig. 1 c, d; Fig 3a and b), most likely in phloem parenchyma cells as was suggested for other members of the Clade III [41]. After floral transition, the GUS expression driven by p*SWEET10:GUS* was dramatically reduced in the leaves and highly detected in the reproductive organs (Additional file 2: Figure S2). This pattern of expression suggests diverse *SWEET10* functions throughout plant development as proposed for some *SWEET* genes [48] In the presence of high levels of FT in the phloem such as in *GAS1:FT* transgenic plants, the expression of *SWEET10* was remarkably increased and restricted to the vasculature (Fig. 1c). This poses the question of how FT activates the transcription of *SWEET10* in the phloem parenchyma cells. One possibility is that *FT* is expressed in this cell type, as suggested for the rice *FT*-like gene *Hd3a* mRNA [49]. However, it was recently shown that in *A. thaliana FT* mRNA is synthesized in specific companion cells of the phloem [15] from where its protein is exported to the sieve elements. In this context, it would be more plausible that the movement of FT and/or SOC1 to the phloem parenchyma cells is responsible for the transcriptional activation of *SWEET10*. Detailed imaging studies of the spatial distribution of FT, SOC1 and SWEET10 using fluorescent markers would contribute to address specifically in which phloem cells they are present. The possibility that SOC1 acts as a mediator of FT-signaling to activate the transcription of *SWEET10* is particularly interesting. Recently, the direct targets of SOC1 were identified using genome-wide approaches [34, 35]. In these studies, the binding of SOC1 to *SWEET10* was not found, suggesting the existence of a third *SWEET10* activating-factor downstream of SOC1 and FT. This factor could be encoded by one of the genes that are highly co-expressed with *SWEET10* such as *SEP3* and *FTM5* (Additional file 2: Figure S1). The identification of transcription factors that bind to *SWEET10* regulatory regions would help understand how the FT-signaling pathway induces the expression of this gene in the vasculature.

### Potential functions of *SWEET10* in regulating flowering

Sugars are believed to promote flowering in several species [50]. In many of these species, floral induction correlates with a rapid increase in the concentration of sucrose in the phloem, especially near the shoot apex. This phenomenon was shown for instance in *Sinapis alba* (white mustard) [51] and *Xanthium strumarium* (rough cocklebur) [52]. In *A. thaliana*, the inductive LD treatment triggers a transient increase of sucrose in the leaf exudate [53]. Moreover, transgenic plants of different species, such as tomato, potato and *A. thaliana*, which over-accumulated sucrose in leaves flowered earlier than the control wild-type plants [53–55]. These results suggest that sucrose acts as a signal during the photoperiod flowering induction. Whether the levels of sucrose or other sugars change during floral transition in the SAM remains unclear, as its quantification in this tissue is technically challenging. Indeed, we did not detect significant changes in the concentrations of sucrose, fructose or glucose in shoot apices of *A. thaliana* plants shifted from SDs to LDs (Additional file 2: Figure S7). However, these apical samples include young leaves, a segment of the apical stem and meristems, so we cannot exclude that local changes in sugar concentration occur. Furthermore, in a previous report, sucrose was found to increase in concentration during the floral transition in shoot apices of plants grown under continuous LDs [6]. Therefore, sugars, and sucrose in particular, could act in the SAM to induce or facilitate the floral transition in response to LDs. In this context, sugar transporters such as SWEET proteins might play an important role in this process. In agreement with this, the overexpression of *SWEET10* in *A. thaliana* triggered a significant acceleration of flowering (Fig. 3a and b). Interestingly, other sugar transporters have also been related to flowering-time control. For example, *A. thaliana* mutants deficient in *SUCROSE TRANSPORTER 9* (*SUC9*) were early flowering under SDs, probably by an increase in the phloem-loading of sucrose [56]. Therefore, the transport of sugars from leaves to the SAM mediated by specialized transporters could contribute to the floral transition in *A. thaliana* and other species. However, the precise role of *SWEET10* in controlling flowering time is still unknown. One possible scenario is that *SWEET10* is transcriptionally induced downstream of FT (and SOC1) in order to supply sugars to the SAM at the time that floral transition occurs. This would contribute to satisfying increased energy requirements of the shoot meristem in order to undergo the increased growth and cell division associated with the floral transition and the initiation of floral organogenesis. Remarkably, the transcription factor CO, which is part of the photoperiodic flowering pathway of Arabidopsis, is responsible for the mobilization of sugars from amylose during the floral transition [57]. Therefore, the photoperiod pathway could affect sugar transport at least at two distinct levels: through CO to mobilize sugars [57] and then through FT to facilitate sugar transport to the SAM. In an alternative scenario, sugars transported by SWEET10 would contribute to the movement of FT towards the SAM. However, so far there is no evidence that sugars are involved in FT transport, although it is proposed to move through the phloem in the photosynthate stream. In both situations, the effect of *SWEET10* overexpression on flowering time would depend on FT activity. In agreement with this, the early-flowering phenotype of *35S:SWEET10* transgenics was suppressed under SD conditions (Fig. 3c). Moreover, the overexpression of *SWEET10* resulted in the induction of genes in the shoot apex related to FT function (Fig. 4). Among them, *SPL4* and *SPL9* that are also known to be upregulated by gibberellin signaling under inductive LD conditions [58]. Interestingly, SWEET proteins were proposed to transport gibberellins as well as sucrose [47], suggesting that FT could regulate both sucrose and gibberellin levels at the apex during flowering by upregulating *SWEET10*. Also, in potato FT was proposed to regulate SWEET function at the post-translational level to prevent leakage of sugar into the apoplast [59].

Overexpression of SWEET10 caused early flowering, but loss of function mutants were not affected in flowering time. Overexpression from the *35S* promoter is widely used to address the function of genes, but loss-of-function genetics would provides more definitive evidence on the role of *SWEET* genes in flowering time-control. *SWEET10* single mutants examined here did not show any striking phenotype related to flowering-time (Fig. 3d and e), which could be explained by functional redundancy between members of the *SWEET* family. *SWEET13* is an obvious candidate to play a redundant function, as its expression was also upregulated in p*GAS1:FT ft-10 tsf-1* plants compared to *ft-10 tsf-1* double mutants (Table 1). However, *SWEET13* overexpression did not result in early flowering (Fig. 5b), and higher order mutants might also show pleiotropic phenotypes. Thus, a systematic study of higher order loss-of-function mutants could be necessary to obtain a more complete picture of the *SWEET* genes function in flowering-time. Furthermore, induction of SWEET proteins during flowering might contribute to the altered metabolic state of the vasculature during floral transition without visibly altering leaf number or flowering time. Nevertheless, the reduced expression of *SWEET10* in *ft tsf* double mutants and its increased expression after transfer to LDs, support a relationship between *SWEET10* transcription and flowering.

## Conclusions

The data shown here indicate that transcriptional activation of *SWEET10* by FT and SOC1 occurs during the promotion of flowering mediated by inductive photoperiod and that overexpression of *SWEET10* causes early flowering consistent with a functional role in this process. This emphasizes the likely significance of changing patterns in sugar transport during the floral transition. Moreover, it supports the idea that FT not only plays a role as a long-distance signaling molecule but that it can also function in leaves to bring about transcriptional changes that eventually contribute to flowering-time regulation in the SAM.

## Methods

### Plant materials

*Arabidopsis thaliana* Columbia-0 (Col) was used as wild-type in all experiments and for plant transformation. The transgenic plants *pGAS1:FT ft-10 tsf-1, pGAS1:FT* and *pGAS1:FT soc1–2* were previously described [11, 60]. The mutant alleles used were *soc1–2* [33] and *ft-10 tsf-1* [11]. The *SWEET10* CDS sequence was obtained from the Arabidopsis Biological Resource Center (http://www.arabidopsis.org/) (clone U15254) and cloned in the pAlligator-2 [61] and p*SUC2:GW* [11] vectors to generate the p*35S:SWEET10* and p*SUC2:SWEET10* lines, respectively. To generate *35S:SW13* and *35S:SW14* transgenic plants, *SWEET13* and *SWEET14* cDNAs were amplified (primer combinations in Additional file 2: Table S1) and inserted into a cloning vector. The inserted sequences were then cloned into the binary vector pBE2113 [62] with XbaI and SmaI restriction sites. The p*SWEET10:GUS* lines were obtained by cloning a 3 Kb region upstream of the transcriptional starting site of the *SWEET10* gene (primers in Additional file 2: Table S1) into the pGreen-GW-GUS vector [63]. For *pSWEET11:GUS* and *pSWEET12:GUS* constructs, promoter regions (approximately 2 kb) of *SWEET11* and *SWEET12* were amplified (primer combinations in Additional file 2: Table S1). The amplified fragments were cloned into pENTR/D-TOPO and then into pGWB3 [64]. *pSWEET13:GUS* and *pSWEET14:GUS* transgenic plants were described previously [47]. T-DNA line SALK_041830 was obtained from the Nottingham Arabidopsis Stock Center (NASC) (http://arabidopsis.info/). For the production of the *SWEET10* silencing lines, a amiRNA targeting this gene was generated by using the online tool WMD3 (primers in Additional file 2: Table S1) and the artificial miRNA vector pRS300 [65]. The resulting *amiRNA-SWEET10* construct was cloned in the vector pAlligator-2. Arabidopsis plants were transformed following the floral dip method [66].

### Plant growth conditions

Seeds were stratified on soil for 3 day in the dark at 4 °C. Plants were grown under controlled environmental conditions at 22 °C and white fluorescent light (150 μmol/m^2^/s), either in LDs (16 h light/8 h dark) or in SDs (8 h light/16 h dark). Flowering time was scored by counting total leaves number (caulines and rosettes) of at least 10 plants per genotype. Each experiment was performed at least twice. For RT-qPCR experiments leaves and shoot apices (containing a segment of the apical stem, SAM and young leaves) were dissected manually.

### Microarray experiment

Col-0, *ft-10 tsf-1* and *pGAS1:FT ft-10 tsf-1* plants were grown under LD conditions during 9 days. Leaves of each genotypes were harvested at the end of the light period (ZT16). RNA from three independent biological replicates was extracted using the RNA Plant Mini kit, QIAGEN (www1.qiagen.com/). The concentration of the total RNA was determined using a NanoDrop ND1000 spectrophotometer. The probe synthesis and the hybridization were performed as previously described in [67]. One microgram of total RNA was reverse transcribed into cDNA using an oligo(dT)-T7 primer, and was then converted into cRNA and linearly amplified by T7 in vitro transcription reaction using the standard Ambion protocol (MessageAmp aRNA Kit, Ambion). cRNA was then reverse transcribed with random primers to dUTP-containing ds cDNA (WT ds cDNA Synthesis Kit, catalog no. 900813; Affymetrix). Fragmentation and labeling was performed with the GeneChip WT double-stranded DNA Terminal Labeling Kit (catalog no. 900812, Affymetrix). After fragmentation, 7.5 μg of ds-cDNA was hybridized for 16 h at 45C on GeneChip Arabidopsis Tiling 1.0R Array. GeneChips were washed and stained with Fluidics Script FS450_0001 in the Affymetrix Fluidics Station 450. Then, the GeneChips were scanned using the GeneChip Scanner 3000 7G. Data was processed in R v2.8.1 using the probe annotation athtiling1.0rcdf as described in [68]. Probe-level data were pre-processed using the RMA algorithm implemented in the Bioconductor package Affy v1.24.2. Linear models and empirical Bayes methods from the Limma package v2.14 of Bioconductor were applied to derive a *P* value, false discovery rate (FDR; P adjusted), and mean of log2-based ratio across replicates. The data were deposited in the Gene Expression Omnibus at the National Center for Biotechnology Information (GEO accession number GSE125054).

### RT-qPCR

RNA expression analyses were performed as described in [69]. The RNA was extracted from plant tissue (leaves or shoot apices) by using the RNeasy Plant Mini Kit (Qiagen) and treated with DNA-free DNase (Ambion). One microgram of total RNA (quantified in a Nanodrop ND-1000) was used for reverse transcription by using the Superscript III (Invitrogen). Levels of gene expression were quantified by qPCR in a LightCycler 480 instrument (Roche) using the *PEX4* gene (*AT5G25760*) as a reference. Three biological replicates were performed for each qRT-PCR assay. The average of the three replicates is shown. The list of primers used for expression analyses can be found in the Additional file 2: Table S1.

### Histochemical staining for GUS activity

Transgenic plants of *pSWEET10:GUS*, *pSWEET11:GUS*, *pSWEET12:GUS*, *pSWEET13:GUS* and *pSWEET14:GUS* were fixed with cold 90% (v/v) acetone for 30 min on ice, then washed with 50 mM sodium phosphate buffer twice. The samples were then immersed in the X-Gluc staining solution [50 mM NaPO_4_ buffer (pH 7.0), 0.5 mM K_3_Fe(CN)_6_, 0.5 mM K_4_Fe(CN)_6_, 0.1% (v/v) Triton X-100, 0.5 mg/ml 5-bromo-4-chloro− 3-indolyl-beta-D-glucuronide (X-Gluc) in H_2_O] under vacuum for 15 min, and then incubated at 37 °C in the dark for 40 h. After the reaction, the samples were washed with 50 mM sodium phosphate buffer, dehydrated through an ethanol series and observed under stereo microscope (Zeiss, Stemi 508).

For histological analysis, the samples were embedded in paraffin, and sliced with the microtome (Leica, RM2125 RTS) to make serial sections of 8-μm thickness. After deparaffinization and rehydration, the sections were observed with the differential interference contrast (DIC) microscope (Zeiss, Axio Imager M2).

### Sugar measurements

For each sample, 30 apexes were harvested and frozen in liquid nitrogen. The samples were extracted in chloroform/methanol/water according to [70]. The aqueous phase was used for sugar measurement and the chloroform phase for protein determination. Sucrose, fructose and glucose were determined photospectrometrically using a 96-well plate reader (Synergy HT from BioTek, U.S.A.) based on the method described in [71] and adapted to the 96-well format by [72]. Protein content was measured according to [73] using the DC™ Protein Assay kit (Bio-Rad Laboratories, U.S.A.) and the values were used for normalization of the sugar data.

## Supplementary information

**Additional file 1.** Results of the expression studies performed on the GeneChip Arabidopsis Tiling 1.0R Array.

**Additional file 2: Table S1.** Primers used in this study. **Figure S1.** In silico analyses of co-expressed gene networks around *SWEET10.* (A) Gene network representation and list of genes correlated to *SWEET10* during development generated by the GENEVESTIGATOR software [74]. (B) The *Arabidopsis thaliana* trans-factor and cis-element prediction database ATTED-II [75], implemented in www.arabidopsis.org, was used to predict and visualize co-expressed genes around *SWEET10*. **Figure S2.** Expression of *pSWEET10:GUS* in adult Arabidopsis plants. (A) GUS expression in a whole plant expressing p*SWEET10:GUS*. The T4 transgenic plant shown in (A) was grown under LDs until siliques were produced. Detail of an inflorescence (B) and a silique (C) showing GUS expression. **Figure S3.** Expression levels of *SWEET10* in T1 transgenic lines overexpressing *SWEET10*. The expression levels of *SWEET10* was quantified in Col-0 and *35S:SWEET10* T1 lines under LDs. Leaves were collected at ZT8. Errors bars indicate Standard Deviation. **Figure S4.** Effect of the overexpression of *SWEET10* from *SUC2* promoter on flowering time under LDs. At least 10 plants were used for each experiment. Asterisk indicates a significant different compared to Col-0 (T-test, *p*-Value ≤0.05). Errors bars indicate Standard Deviation. **Figure S5.** Analysis of *amiR*-*SWEET10* transgenic plants. (A) Flowering time of 44 *amiR-SWEET10* T1 lines compared to Col-0 under LDs. TLN: Total Leaf Number. (B) *SWEET10* expression levels in a subset of T3 *amiR-SWEET10* lines. **Figure S6.** Photoperiod-dependent expression profile of *SWEET11, 12, 13* and *14.* GUS expression in plants expressing p*SWEET10:GUS*. Plants were grown under SDs for 2 weeks, shifted to LDs and collected for GUS staining at ZT8 in different days. Scale bar = 5 mm. **Figure S7.** Levels of sugar during the photoperiodic induction of flowering. Col-0 and *ft tsf* plants were grown under SDs for 2 weeks, shifted to LDs shoot apices were harvested at ZT8 in different days. Shoot apices were harvested and used to quantify the concentration of fructose (fru), glucose (glu) and sucrose (suc). Errors bars indicate Standard Deviation.

## Data Availability

The transcriptomic datasets generated during the current study are available in the GEO NCBI repository under the accession number GSE125054 and in the supplementary information files. *Arabidopsis thaliana* Columbia, which was used throughout this work, is available from the Arabidopsis Stock Centre, Nottingham University, UK or the Arabidopsis Biological Resource Centre, Ohio State University or from the authors. No permissions were required to use *Arabidopsis thaliana*. All other plants materials are available from the corresponding authors.

## Abbreviations

FDR: False discovery rate
LDs: Long days
RT-qPCR: Quantitative reverse transcription polymerase chain reaction
SAM: Shoot apical meristem
SDs: Short days
