# Supplementary material for: The sugar transporter SWEET10 acts downstream of FLOWERING LOCUS T during floral transition of Arabidopsis thaliana
Source: BMC Plant Biol. 2020 Feb 3;20:53. doi: 10.1186/s12870-020-2266-0 (PMC6998834; doi:10.1186/s12870-020-2266-0)
Supplement: Supplementary file 2 — Additional file 2: Table S1. Primers used in this study. Figure S1. In silico analyses of co-expressed gene networks around SWEET10. (A) Gene network representation and list of genes correlated to SWEET10 during development generated by the GENEVESTIGATOR software [74]. (B) The Arabidopsis thaliana trans-factor and cis-element prediction database ATTED-II [75], implemented in www.arabidopsis.org, was used to predict and visualize co-expressed genes around SWEET10. Figure S2. Expression of pSWEET10:GUS in adult Arabidopsis plants. (A) GUS expression in a whole plant expressing pSWEET10:GUS. The T4 transgenic plant shown in (A) was grown under LDs until siliques were produced. Detail of an inflorescence (B) and a silique (C) showing GUS expression. Figure S3. Expression levels of SWEET10 in T1 transgenic lines overexpressing SWEET10. The expression levels of SWEET10 was quantified in Col-0 and 35S:SWEET10 T1 lines under LDs. Leaves were collected at ZT8. Errors bars indicate Standard Deviation. Figure S4. Effect of the overexpression of SWEET10 from SUC2 promoter on flowering time under LDs. At least 10 plants were used for each experiment. Asterisk indicates a significant different compared to Col-0 (T-test, p-Value ≤0.05). Errors bars indicate Standard Deviation. Figure S5. Analysis of amiR-SWEET10 transgenic plants. (A) Flowering time of 44 amiR-SWEET10 T1 lines compared to Col-0 under LDs. TLN: Total Leaf Number. (B) SWEET10 expression levels in a subset of T3 amiR-SWEET10 lines. Figure S6. Photoperiod-dependent expression profile of SWEET11, 12, 13 and 14. GUS expression in plants expressing pSWEET10:GUS. Plants were grown under SDs for 2 weeks, shifted to LDs and collected for GUS staining at ZT8 in different days. Scale bar = 5 mm. Figure S7. Levels of sugar during the photoperiodic induction of flowering. Col-0 and ft tsf plants were grown under SDs for 2 weeks, shifted to LDs shoot apices were harvested at ZT8 in different days. Shoot apices were harvested an [file 12870_2020_2266_MOESM2_ESM.zip › Additional file 2-Revised.docx]

**The sugar transporter SWEET10 acts downstream of *FLOWERING LOCUS T* during floral transition of *Arabidopsis thaliana***

Fernando Andrés^1,4^§, Atsuko Kinoshita^1,4^, Naveen Kalluri^1^, Virginia Fernández^1^, Tiago MD Cruz^1^, Seonghoe Jang^1^, Yasutaka Chiba^2^, Mitsunori Seo^2^, Tabea Mettler-Altmann^3^, Bruno Huettel^1^ and George Coupland^1^§

^1^ Max Planck Institute for Plant Breeding Research, Carl-von-Linne-Weg 10, D50829, Germany

^2^ RIKEN Center for Sustainable Resource Science, 1-7-22 Suehiro-cho, Tsurumi-ku, Yokohama, Kanagawa 230-0045, JAPAN

^3^Cluster of Excellence on Plant Sciences and Institute of Plant Biochemistry, Heinrich-Heine University, 40225 Düsseldorf, Germany

^4^ Contributed equally to this work.

§Corresponding authors. E-mail: [fernando.andres-lalaguna@inra.fr](mailto:fernando.andres-lalaguna@inra.fr) and [coupland@mpipz.mpg.de](mailto:coupland@mpipz.mpg.de)

**Supplementary data**

**Table S1 2**

**Figure S1 Legend 3**

**Figure S2 Legend 4**

**Figure S3 Legend 5**

**Figure S4 Legend 6**

**Figure S5 Legend 7**

**Figure S6 Legend 8**

**Figure S7 Legend 9**

**Table S1. Primers used in this study.**

| **Gene** | **Forward** | **Reverse** |
| --- | --- | --- |
| **RT-qPCR** | | |
| SWEET10 | CGCAATCTTCGTGTTGACAT | AGAGACCGAATGGCATGAAC |
| FT | tggtgactgatatccctgct | accctggtgcatacactgtt |
| SOC1 | TGATGAAGAGAGTAGCCCAAG | TGAGAGAGAGAGAGTGAGAGAGAAA |
| SPL4 | CATCATTCAAGCGACCACAG | TTGGCAAGGAAAAGCTAGGA |
| FD | TCAACCTTGCTTCCATCC | GGTTTTGGTTGTGGTGGTTT |
| FUL | TTGCAAGATCACAACAATTCGCTTCTC | GAGAGTTTGGTTCCGTCAACGACGATG |
| SPL9 | GGCTGGTATCGAACAGAGGT | CAAATTCCGGAAGCTGATG |
| MIR156C | GGTAGCCACCCCTTTCAAAT | GGTAGCCACCCCTTTCAAAT |
| PEX4 | TTACGAAGGCGGTGTTTTTC | GGCGAGGCGTGTATACATTT |
| **Cloning** | | |
| SWEET10 promoter | GTTGATAACGACGACGACG | CACATTTTGAGGAAGCTAATC |
| SWEET11 promoter | CACCCATAGATAAAAAGACTTTGACAAACTATAC | GGTATATAAGTTGTTAGGAGAAGGAA |
| SWEET12 promoter | CACCGGTAATTAACACTCCTGTATCTAC | GAATGTCGATCTTTGGTTCCG |
| SWEET13 cDNA | TCTAGAATGGCTCTAACTAACAATTTATGGGC | CCCGGGTTAAACTTGACTTTGTTTCTGGACATCC |
| SWEET14 cDNA | TCTAGAATGGTTCTCACTCACAACGTATTG | CCCGGGTTAGTTTGGCATTTTCTTGTCCATCTG |
| **Cloning an artificial microRNA against SWEET10** | | |
| amiR-SWEET10 I miR-s | gaTGTAGACGATTTGTACGGCAAtctctcttttgtattcc |  |
| amiR-SWEET10 II miR-a |  | gaTTGCCGTACAAATCGTCTACAtcaaagagaatcaatga |
| amiR-SWEET10 III miR*s | gaTTACCGTACAAATGGTCTACTtcacaggtcgtgatatg |  |
| amiR-SWEET10 IV miR*a |  | gaAGTAGACCATTTGTACGGTAAtctacatatatattcct |

**Figure S1. *In silico* analyses of co-expressed gene networks around *SWEET10.*** (A) Gene network representation and list of genes correlated to *SWEET10* during development generated by the GENEVESTIGATOR software [73]. (B) The *Arabidopsis thaliana* trans-factor and cis-element prediction database ATTED-II [74], implemented in [www.arabidopsis.org](http://www.arabidopsis.org/), was used to predict and visualize co-expressed genes around *SWEET10*.

**Figure S2. Expression of *pSWEET10:GUS* in adult Arabidopsis plants.** (A) GUS expression in a whole plant expressing p*SWEET10:GUS*. The T4 transgenic plant shown in (A) was grown under LDs until siliques were produced. Detail of an inflorescence (B) and a silique (C) showing GUS expression.

**Figure S3. Expression levels of *SWEET10* in T1 transgenic lines overexpressing *SWEET10*.** The expression levels of *SWEET10* was quantified in Col-0 and *35S:SWEET10* T1 lines under LDs. Leaves were collected at ZT8. Errors bars indicate Standard Deviation.

**Figure S4. Effect of the overexpression of *SWEET10* from *SUC2* promoter on flowering time under LDs.** At least 10 plants were used for each experiment. Asterisk indicates a significant different compared to Col-0 (T-test, p-Value ≤ 0.05). Errors bars indicate Standard Deviation.

**Figure S5. Analysis of *amiR*-*SWEET10* transgenic plants.** (A) Flowering time of 44 *amiR-SWEET10* T1 lines compared to Col-0 under LDs. TLN: Total Leaf Number. (B) *SWEET10* expression levels in a subset of T3 *amiR-SWEET10* lines.

**Figure S6. Photoperiod-dependent expression profile of *SWEET11, 12, 13* and *14.*** GUS expression in plants expressing p*SWEET10:GUS*. Plants were grown under SDs for two weeks, shifted to LDs and collected for GUS staining at ZT8 in different days. Scale bar = 5 mm.

**Figure S7. Levels of sugar during the photoperiodic induction of flowering.** Col-0 *and ft tsf* plants were grown under SDs for two weeks, shifted to LDs shoot apices were harvested at ZT8 in different days. Shoot apices were harvested and used to quantify the concentration of fructose (fru), glucose (glu) and sucrose (suc).
