# Supplementary figures and images for: The sugar transporter SWEET10 acts downstream of FLOWERING LOCUS T during floral transition of Arabidopsis thaliana
Source: BMC Plant Biol. 2020 Feb 3;20:53. doi: 10.1186/s12870-020-2266-0 (PMC6998834; doi:10.1186/s12870-020-2266-0)

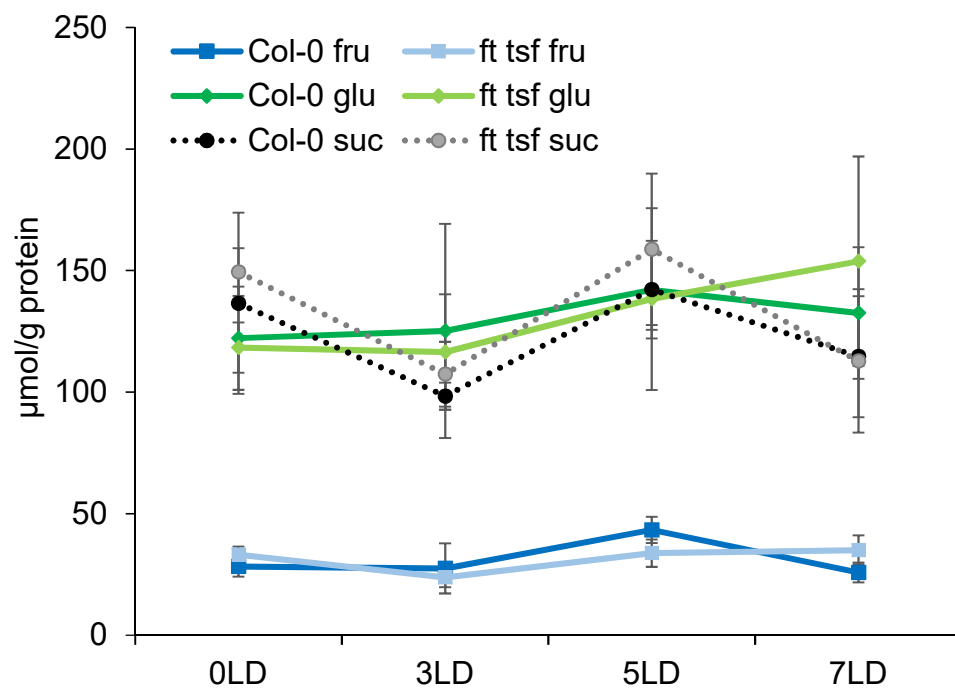

Figure S7

Supplement: Supplementary file 2 — Additional file 2: Table S1. Primers used in this study. Figure S1. In silico analyses of co-expressed gene networks around SWEET10. (A) Gene network representation and list of genes correlated to SWEET10 during development generated by the GENEVESTIGATOR software [74]. (B) The Arabidopsis thaliana trans-factor and cis-element prediction database ATTED-II [75], implemented in www.arabidopsis.org, was used to predict and visualize co-expressed genes around SWEET10. Figure S2. Expression of pSWEET10:GUS in adult Arabidopsis plants. (A) GUS expression in a whole plant expressing pSWEET10:GUS. The T4 transgenic plant shown in (A) was grown under LDs until siliques were produced. Detail of an inflorescence (B) and a silique (C) showing GUS expression. Figure S3. Expression levels of SWEET10 in T1 transgenic lines overexpressing SWEET10. The expression levels of SWEET10 was quantified in Col-0 and 35S:SWEET10 T1 lines under LDs. Leaves were collected at ZT8. Errors bars indicate Standard Deviation. Figure S4. Effect of the overexpression of SWEET10 from SUC2 promoter on flowering time under LDs. At least 10 plants were used for each experiment. Asterisk indicates a significant different compared to Col-0 (T-test, p-Value ≤0.05). Errors bars indicate Standard Deviation. Figure S5. Analysis of amiR-SWEET10 transgenic plants. (A) Flowering time of 44 amiR-SWEET10 T1 lines compared to Col-0 under LDs. TLN: Total Leaf Number. (B) SWEET10 expression levels in a subset of T3 amiR-SWEET10 lines. Figure S6. Photoperiod-dependent expression profile of SWEET11, 12, 13 and 14. GUS expression in plants expressing pSWEET10:GUS. Plants were grown under SDs for 2 weeks, shifted to LDs and collected for GUS staining at ZT8 in different days. Scale bar = 5 mm. Figure S7. Levels of sugar during the photoperiodic induction of flowering. Col-0 and ft tsf plants were grown under SDs for 2 weeks, shifted to LDs shoot apices were harvested at ZT8 in different days. Shoot apices were harvested an [file 12870_2020_2266_MOESM2_ESM.zip › Figure S7.pdf]

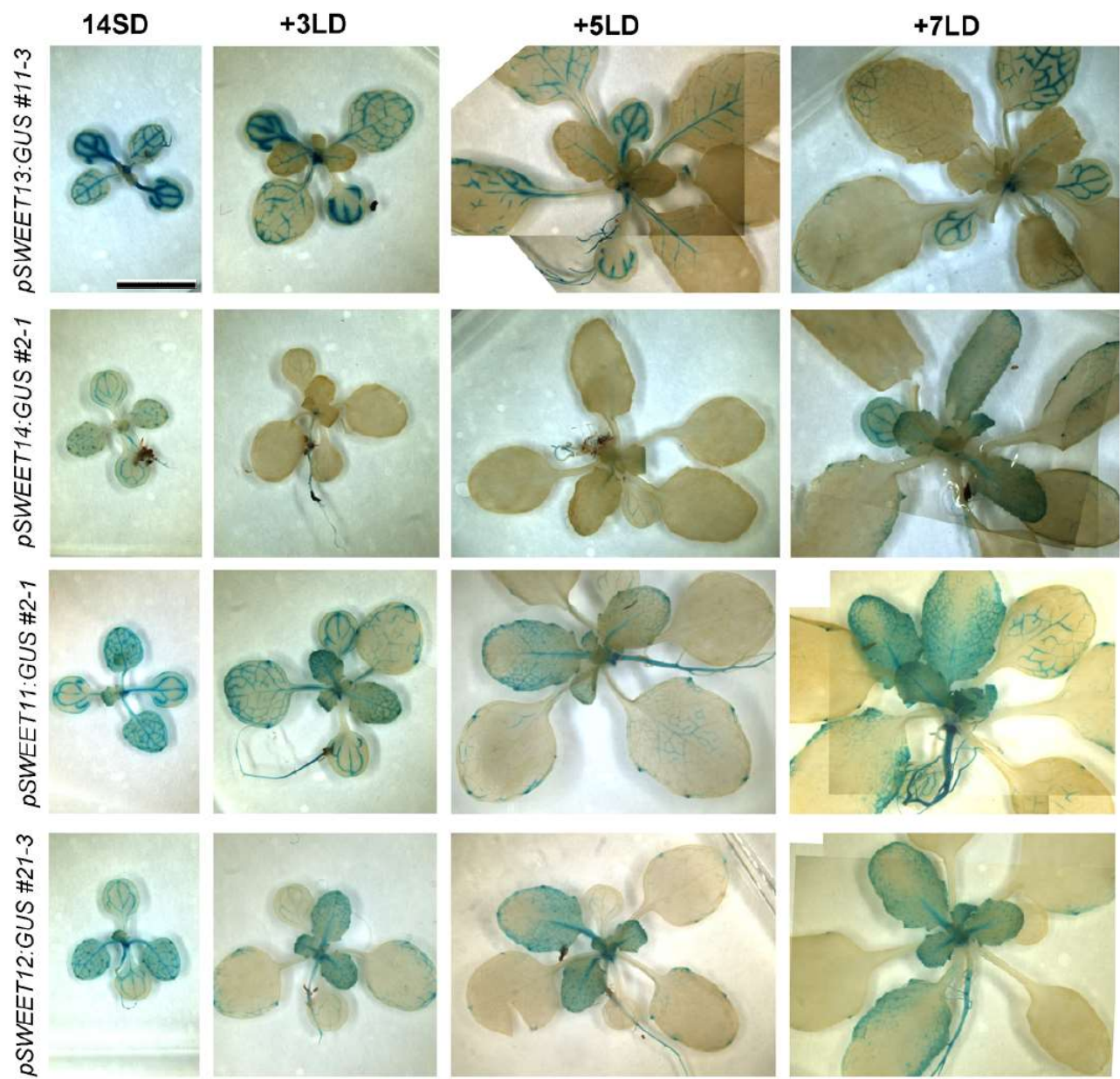

Figure S6

Supplement: Supplementary file 2 — Additional file 2: Table S1. Primers used in this study. Figure S1. In silico analyses of co-expressed gene networks around SWEET10. (A) Gene network representation and list of genes correlated to SWEET10 during development generated by the GENEVESTIGATOR software [74]. (B) The Arabidopsis thaliana trans-factor and cis-element prediction database ATTED-II [75], implemented in www.arabidopsis.org, was used to predict and visualize co-expressed genes around SWEET10. Figure S2. Expression of pSWEET10:GUS in adult Arabidopsis plants. (A) GUS expression in a whole plant expressing pSWEET10:GUS. The T4 transgenic plant shown in (A) was grown under LDs until siliques were produced. Detail of an inflorescence (B) and a silique (C) showing GUS expression. Figure S3. Expression levels of SWEET10 in T1 transgenic lines overexpressing SWEET10. The expression levels of SWEET10 was quantified in Col-0 and 35S:SWEET10 T1 lines under LDs. Leaves were collected at ZT8. Errors bars indicate Standard Deviation. Figure S4. Effect of the overexpression of SWEET10 from SUC2 promoter on flowering time under LDs. At least 10 plants were used for each experiment. Asterisk indicates a significant different compared to Col-0 (T-test, p-Value ≤0.05). Errors bars indicate Standard Deviation. Figure S5. Analysis of amiR-SWEET10 transgenic plants. (A) Flowering time of 44 amiR-SWEET10 T1 lines compared to Col-0 under LDs. TLN: Total Leaf Number. (B) SWEET10 expression levels in a subset of T3 amiR-SWEET10 lines. Figure S6. Photoperiod-dependent expression profile of SWEET11, 12, 13 and 14. GUS expression in plants expressing pSWEET10:GUS. Plants were grown under SDs for 2 weeks, shifted to LDs and collected for GUS staining at ZT8 in different days. Scale bar = 5 mm. Figure S7. Levels of sugar during the photoperiodic induction of flowering. Col-0 and ft tsf plants were grown under SDs for 2 weeks, shifted to LDs shoot apices were harvested at ZT8 in different days. Shoot apices were harvested an [file 12870_2020_2266_MOESM2_ESM.zip › Figure S6.pdf]

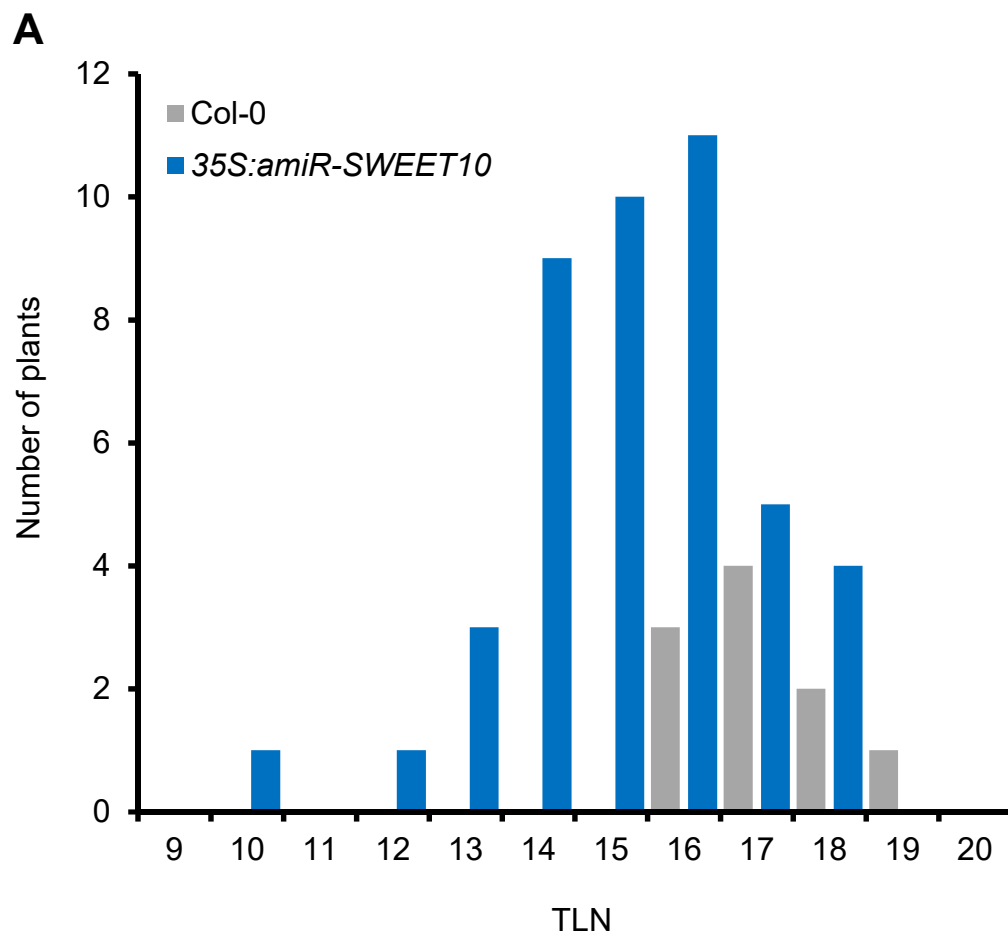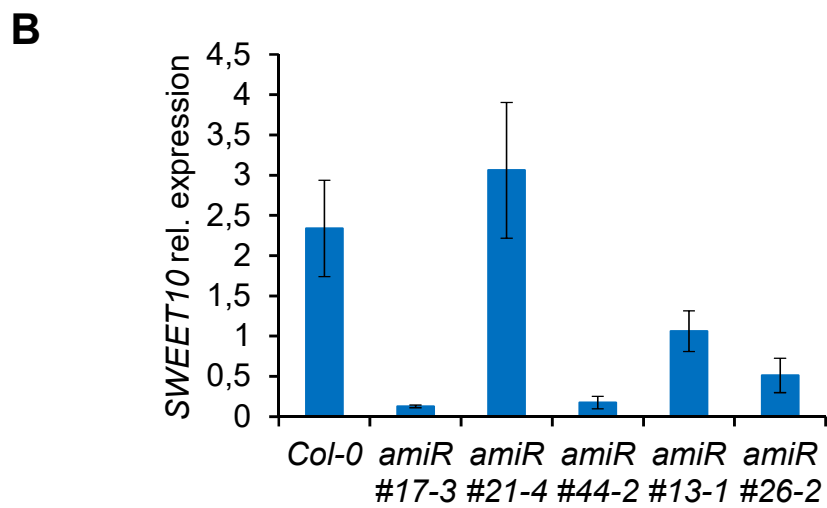

Figure S5

Supplement: Supplementary file 2 — Additional file 2: Table S1. Primers used in this study. Figure S1. In silico analyses of co-expressed gene networks around SWEET10. (A) Gene network representation and list of genes correlated to SWEET10 during development generated by the GENEVESTIGATOR software [74]. (B) The Arabidopsis thaliana trans-factor and cis-element prediction database ATTED-II [75], implemented in www.arabidopsis.org, was used to predict and visualize co-expressed genes around SWEET10. Figure S2. Expression of pSWEET10:GUS in adult Arabidopsis plants. (A) GUS expression in a whole plant expressing pSWEET10:GUS. The T4 transgenic plant shown in (A) was grown under LDs until siliques were produced. Detail of an inflorescence (B) and a silique (C) showing GUS expression. Figure S3. Expression levels of SWEET10 in T1 transgenic lines overexpressing SWEET10. The expression levels of SWEET10 was quantified in Col-0 and 35S:SWEET10 T1 lines under LDs. Leaves were collected at ZT8. Errors bars indicate Standard Deviation. Figure S4. Effect of the overexpression of SWEET10 from SUC2 promoter on flowering time under LDs. At least 10 plants were used for each experiment. Asterisk indicates a significant different compared to Col-0 (T-test, p-Value ≤0.05). Errors bars indicate Standard Deviation. Figure S5. Analysis of amiR-SWEET10 transgenic plants. (A) Flowering time of 44 amiR-SWEET10 T1 lines compared to Col-0 under LDs. TLN: Total Leaf Number. (B) SWEET10 expression levels in a subset of T3 amiR-SWEET10 lines. Figure S6. Photoperiod-dependent expression profile of SWEET11, 12, 13 and 14. GUS expression in plants expressing pSWEET10:GUS. Plants were grown under SDs for 2 weeks, shifted to LDs and collected for GUS staining at ZT8 in different days. Scale bar = 5 mm. Figure S7. Levels of sugar during the photoperiodic induction of flowering. Col-0 and ft tsf plants were grown under SDs for 2 weeks, shifted to LDs shoot apices were harvested at ZT8 in different days. Shoot apices were harvested an [file 12870_2020_2266_MOESM2_ESM.zip › Figure S5.pdf]

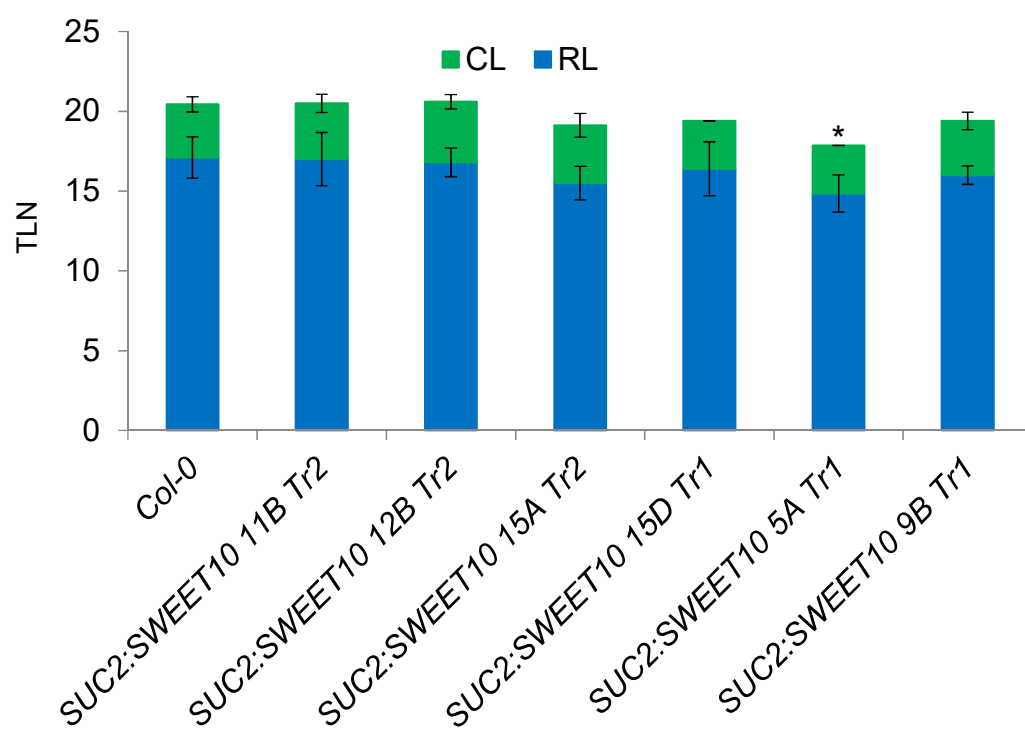

Figure S4

Supplement: Supplementary file 2 — Additional file 2: Table S1. Primers used in this study. Figure S1. In silico analyses of co-expressed gene networks around SWEET10. (A) Gene network representation and list of genes correlated to SWEET10 during development generated by the GENEVESTIGATOR software [74]. (B) The Arabidopsis thaliana trans-factor and cis-element prediction database ATTED-II [75], implemented in www.arabidopsis.org, was used to predict and visualize co-expressed genes around SWEET10. Figure S2. Expression of pSWEET10:GUS in adult Arabidopsis plants. (A) GUS expression in a whole plant expressing pSWEET10:GUS. The T4 transgenic plant shown in (A) was grown under LDs until siliques were produced. Detail of an inflorescence (B) and a silique (C) showing GUS expression. Figure S3. Expression levels of SWEET10 in T1 transgenic lines overexpressing SWEET10. The expression levels of SWEET10 was quantified in Col-0 and 35S:SWEET10 T1 lines under LDs. Leaves were collected at ZT8. Errors bars indicate Standard Deviation. Figure S4. Effect of the overexpression of SWEET10 from SUC2 promoter on flowering time under LDs. At least 10 plants were used for each experiment. Asterisk indicates a significant different compared to Col-0 (T-test, p-Value ≤0.05). Errors bars indicate Standard Deviation. Figure S5. Analysis of amiR-SWEET10 transgenic plants. (A) Flowering time of 44 amiR-SWEET10 T1 lines compared to Col-0 under LDs. TLN: Total Leaf Number. (B) SWEET10 expression levels in a subset of T3 amiR-SWEET10 lines. Figure S6. Photoperiod-dependent expression profile of SWEET11, 12, 13 and 14. GUS expression in plants expressing pSWEET10:GUS. Plants were grown under SDs for 2 weeks, shifted to LDs and collected for GUS staining at ZT8 in different days. Scale bar = 5 mm. Figure S7. Levels of sugar during the photoperiodic induction of flowering. Col-0 and ft tsf plants were grown under SDs for 2 weeks, shifted to LDs shoot apices were harvested at ZT8 in different days. Shoot apices were harvested an [file 12870_2020_2266_MOESM2_ESM.zip › Figure S4.pdf]

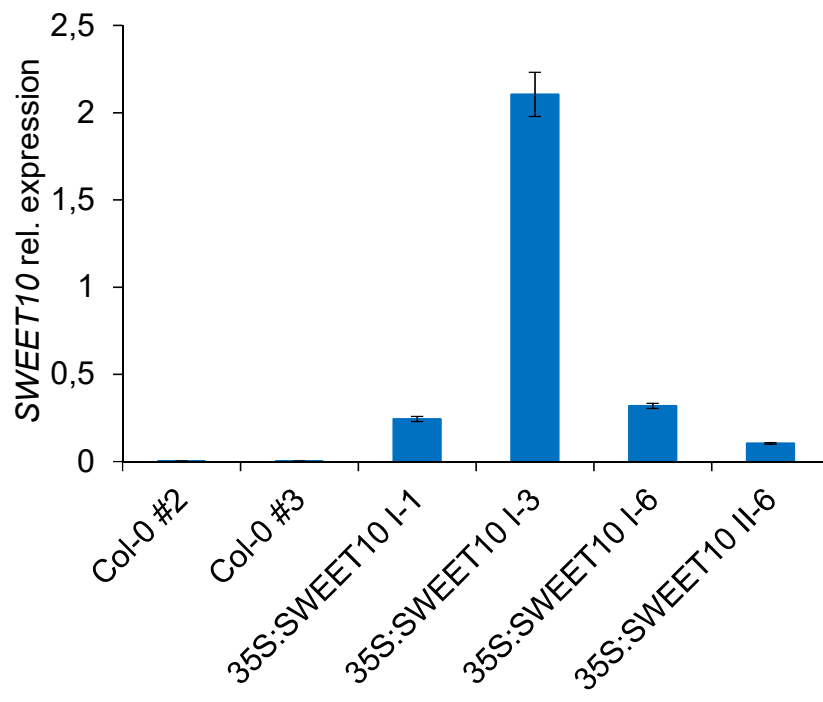

Figure S3

Supplement: Supplementary file 2 — Additional file 2: Table S1. Primers used in this study. Figure S1. In silico analyses of co-expressed gene networks around SWEET10. (A) Gene network representation and list of genes correlated to SWEET10 during development generated by the GENEVESTIGATOR software [74]. (B) The Arabidopsis thaliana trans-factor and cis-element prediction database ATTED-II [75], implemented in www.arabidopsis.org, was used to predict and visualize co-expressed genes around SWEET10. Figure S2. Expression of pSWEET10:GUS in adult Arabidopsis plants. (A) GUS expression in a whole plant expressing pSWEET10:GUS. The T4 transgenic plant shown in (A) was grown under LDs until siliques were produced. Detail of an inflorescence (B) and a silique (C) showing GUS expression. Figure S3. Expression levels of SWEET10 in T1 transgenic lines overexpressing SWEET10. The expression levels of SWEET10 was quantified in Col-0 and 35S:SWEET10 T1 lines under LDs. Leaves were collected at ZT8. Errors bars indicate Standard Deviation. Figure S4. Effect of the overexpression of SWEET10 from SUC2 promoter on flowering time under LDs. At least 10 plants were used for each experiment. Asterisk indicates a significant different compared to Col-0 (T-test, p-Value ≤0.05). Errors bars indicate Standard Deviation. Figure S5. Analysis of amiR-SWEET10 transgenic plants. (A) Flowering time of 44 amiR-SWEET10 T1 lines compared to Col-0 under LDs. TLN: Total Leaf Number. (B) SWEET10 expression levels in a subset of T3 amiR-SWEET10 lines. Figure S6. Photoperiod-dependent expression profile of SWEET11, 12, 13 and 14. GUS expression in plants expressing pSWEET10:GUS. Plants were grown under SDs for 2 weeks, shifted to LDs and collected for GUS staining at ZT8 in different days. Scale bar = 5 mm. Figure S7. Levels of sugar during the photoperiodic induction of flowering. Col-0 and ft tsf plants were grown under SDs for 2 weeks, shifted to LDs shoot apices were harvested at ZT8 in different days. Shoot apices were harvested an [file 12870_2020_2266_MOESM2_ESM.zip › Figure S3.pdf]

**A**

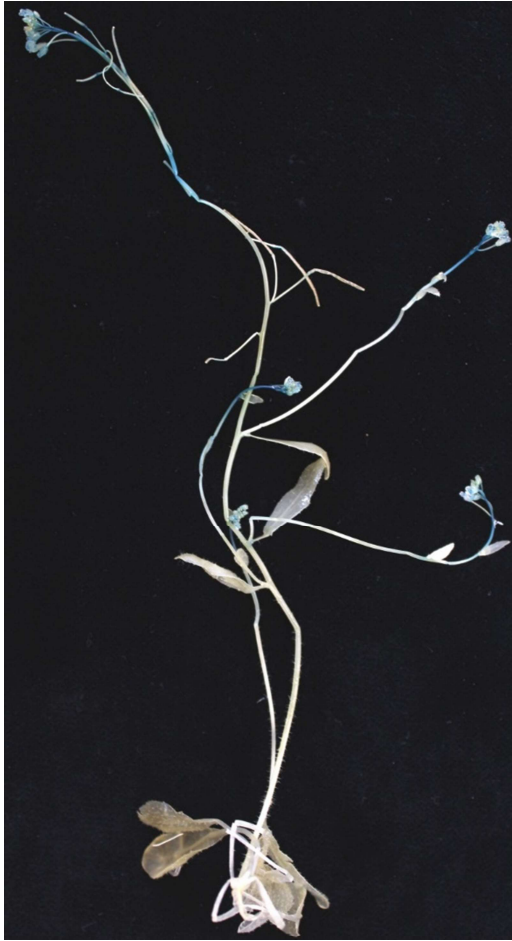

**B**

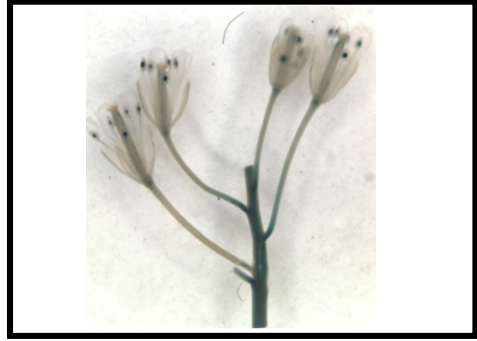

**C**

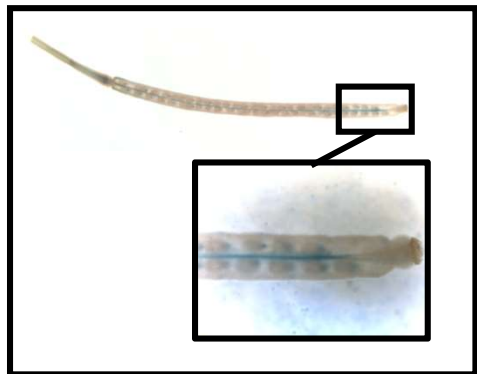

Figure S2

Supplement: Supplementary file 2 — Additional file 2: Table S1. Primers used in this study. Figure S1. In silico analyses of co-expressed gene networks around SWEET10. (A) Gene network representation and list of genes correlated to SWEET10 during development generated by the GENEVESTIGATOR software [74]. (B) The Arabidopsis thaliana trans-factor and cis-element prediction database ATTED-II [75], implemented in www.arabidopsis.org, was used to predict and visualize co-expressed genes around SWEET10. Figure S2. Expression of pSWEET10:GUS in adult Arabidopsis plants. (A) GUS expression in a whole plant expressing pSWEET10:GUS. The T4 transgenic plant shown in (A) was grown under LDs until siliques were produced. Detail of an inflorescence (B) and a silique (C) showing GUS expression. Figure S3. Expression levels of SWEET10 in T1 transgenic lines overexpressing SWEET10. The expression levels of SWEET10 was quantified in Col-0 and 35S:SWEET10 T1 lines under LDs. Leaves were collected at ZT8. Errors bars indicate Standard Deviation. Figure S4. Effect of the overexpression of SWEET10 from SUC2 promoter on flowering time under LDs. At least 10 plants were used for each experiment. Asterisk indicates a significant different compared to Col-0 (T-test, p-Value ≤0.05). Errors bars indicate Standard Deviation. Figure S5. Analysis of amiR-SWEET10 transgenic plants. (A) Flowering time of 44 amiR-SWEET10 T1 lines compared to Col-0 under LDs. TLN: Total Leaf Number. (B) SWEET10 expression levels in a subset of T3 amiR-SWEET10 lines. Figure S6. Photoperiod-dependent expression profile of SWEET11, 12, 13 and 14. GUS expression in plants expressing pSWEET10:GUS. Plants were grown under SDs for 2 weeks, shifted to LDs and collected for GUS staining at ZT8 in different days. Scale bar = 5 mm. Figure S7. Levels of sugar during the photoperiodic induction of flowering. Col-0 and ft tsf plants were grown under SDs for 2 weeks, shifted to LDs shoot apices were harvested at ZT8 in different days. Shoot apices were harvested an [file 12870_2020_2266_MOESM2_ESM.zip › Figure S2.pdf]
